# Transcriptome profiling reveals the developmental regulation of NaCl-treated *Forcipomyia taiwana* eggs

**DOI:** 10.1186/s12864-021-08096-x

**Published:** 2021-11-03

**Authors:** Mu-En Chen, Mong-Hsun Tsai, Hsiang-Ting Huang, Ching-Chu Tsai, Mei-Ju Chen, Da-Syuan Yang, Teng-Zhi Yang, John Wang, Rong-Nan Huang

**Affiliations:** 1 Department of Entomology and Research Center for Plant Medicine, College of Bioresources and Agriculture, National Taiwan University, Taipei, 10617 Taiwan; 2 Biodiversity Research Center, Academia Sinica, Taipei, 11529 Taiwan; 3 Institute of Biotechnology, College of Bioresources and Agriculture, National Taiwan University, Taipei, 10617 Taiwan; 4 Centers for Genomics and Precision Medicine, National Taiwan University, Taipei, 10617 Taiwan; 5 Agricultural Biotechnology Research Center, Academia Sinica, Taipei, 11529 Taiwan

**Keywords:** *Forcipomyia taiwana*, Biting midge, RNA-seq, Melanin, Embryo

## Abstract

**Background:**

The biting midge, *Forcipomyia taiwana,* is one of the most annoying blood-sucking pests in Taiwan. Current chemical control methods only target the adult, not the immature stages (egg to pupa), of *F. taiwana*. Discovering new or alternative tactics to enhance or replace existing methods are urgently needed to improve the effectiveness of *F. taiwana* control. The egg is the least understood life stage in this pest species but may offer a novel point of control as addition of NaCl to the egg environment inhibits development. Thus, the objective of this study was to use RNA profiling to better understand the developmental differences between wild-type melanized (black) and NaCl-induced un-melanized (pink), infertile *F. taiwana* eggs.

**Results:**

After de novo assembly with Trinity, 87,415 non-redundant transcripts (Ft-nr) with an N50 of 1099 were obtained. Of these, 26,247 (30%) transcripts were predicted to have long open reading frames (ORFs, defined here as ≥300 nt) and 15,270 (17.5%) transcripts have at least one predicted functional domain. A comparison between two biological replicates each of black and pink egg samples, although limited in sample size, revealed 5898 differentially expressed genes (DEGs; 40.9% of the transcripts with long ORFs) with ≥2-fold difference. Of these, 2030 were annotated to a Gene Ontology biological process and along with gene expression patterns can be separated into 5 clusters. KEGG pathway analysis revealed that 1589 transcripts could be assigned to 18 significantly enriched pathways in 2 main categories (metabolism and environmental information processing). As expected, most (88.32%) of these DEGs were down-regulated in the pink eggs. Surprisingly, the majority of genes associated with the pigmentation GO term were up-regulated in the pink egg samples. However, the two key terminal genes of the melanin synthesis pathway, *laccase2* and *DCE/yellow*, were significantly down-regulated, and further verified by qRT-PCR.

**Conclusion:**

We have assembled and annotated the first egg transcriptome for *F. taiwana,* a biting midge. Our results suggest that down-regulation of the *laccase2* and *DCE/yellow* genes might be the mechanism responsible for the NaCl-induced inhibition of melanization of *F. taiwana* eggs.

**Supplementary Information:**

The online version contains supplementary material available at 10.1186/s12864-021-08096-x.

## Background

Over 6200 species of biting midges (*Ceratopogonidae*, *Diptera*) in 112 genera have been described worldwide [1] and some of them can be severe biting pests of humans, pets, livestock, and wildlife. Although they are minute to tiny, their bite can inflict a burning sensation which results in different reactions in humans, ranging from a small reddish welt at the bite site to strong local allergic reactions accompanied by significant itching [2]. Moreover, the blood-sucking habits of biting midges raises concerns about a possible involvement in the transmission of disease agents. In particular, *Culicoides* biting midges have been reported as important vectors for numerous animal-associated pathogens, such as African horse sickness virus [3], epizootic hemorrhagic disease virus [4], vesicular stomatitis virus [5], Schmallenberg virus [6], and bluetongue virus [7], as well as the transmission of Oropouche virus which causes acute disease in humans [8].

The most annoying blood-sucking midge in Taiwan is *Forcipomyia taiwana,* which is classified into the subgenus of *Lasiohelea* (*Ceratopogonidae*, *Diptera*). Its bite causes intense itching (or pruitis) and swelling in sensitive individuals [2]. Although there is no evidence that *F. taiwana* transmits human diseases, it accepts only human blood [9] rendering it the most important blood-sucking pest in Taiwan. Blood feeding of *F. taiwana* only occurs during the day, coincident with human activity. Consequently, the outbreak and spread of this midge have resulted in serious disturbances to residents in central Taiwan and even poses a significant impact on resort areas where they drive away visitors. Control options for *F. taiwana* are very limited. The primary options focus on environmental management; however, synthetic pesticides are still the most common strategy for controlling this pest during an outbreak, since they kill adults rapidly and are readily available at local retailers. Unfortunately, control of *F. taiwana* by synthetic insecticides has had only limited success in Taiwan and is coupled with the drawback that chemical insecticides harm both human health and ecological environments. Moreover, current chemical control only targets the adult, not the immature stages (egg to pupa) of *F. taiwana*. Therefore, discovering new or alternative tactics to enhance or replace existing methods are urgently needed to improve the effectiveness of *F. taiwana* control.

**Fig. 1:**
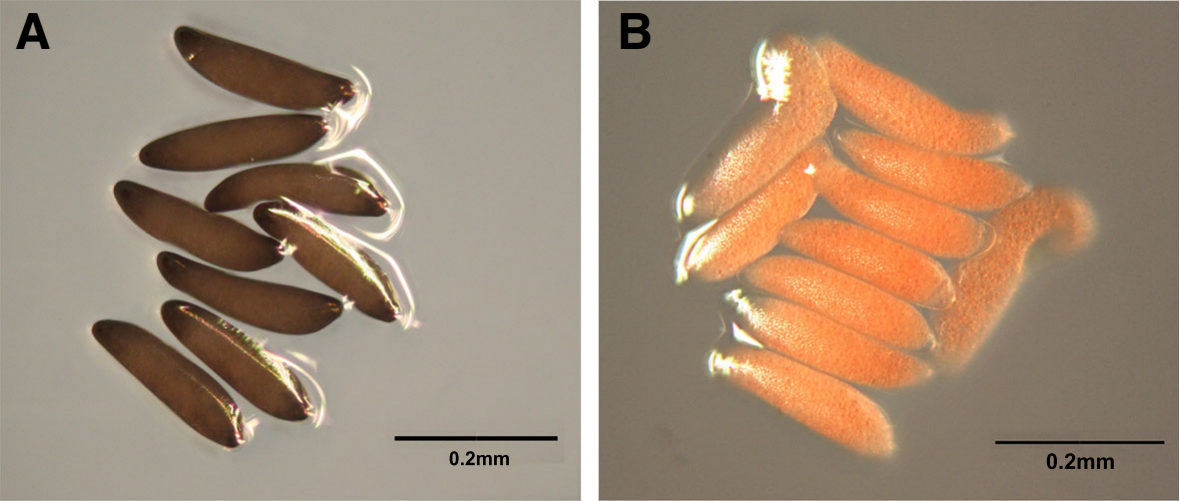
Color of the *F. taiwana* eggs laid in different milieu. **A** *F. taiwana* eggs laid on agar plates without salts melanized normally (black color), **B** while those laid on agar plates containing 0.25 M NaCl did not melanize and remained pink

The egg is the first stage in the insect life cycle. Eliminating eggs, such as through environmental management, is often the simplest and most effective way for pest control, since such strategies produce the strongest results without the use of synthetic insecticides. However, the immature stages of biting midges (in particular the eggs) are far less well understood than the adult stage [10, 11]. Consequently, most attention for managing the biting midge has been given to the adult stage, whereas limited studies on the management of eggs have been conducted. *F. taiwana* embryos are sensitive to humidity and their immotility render them the most vulnerable stage that can be targeted for control. In an attempt to explore treatments that are effective for *F. taiwana* control with minimal adverse effects for humans or the environment, we fortuitously found that the eggs of *F. taiwana* often did not undergo normal darkening of color (i.e., remained pink) and were inviable when laid in salt-containing milieu (Fig. 1).

Chorion darkening, or melanization, protects insect eggs from desiccation and UV damage, and thus, is critical for post-embryonic development and survival in the environment [12]. *F. taiwana* eggs are initially pink and are usually laid in a milieu containing algae. The endochorion of the pink egg begins melanizing within 1 h after being laid, a morphological change similar to that of mosquito eggs. Melanization occurs through the production of eumelanin, a brown to black pigment [13]. In mosquitoes, the canonical melanization pathway genes (*tyrosine 3-monooxygenase (TH), phenoloxidase (PO), Dopa decarboxylase (DDC), laccase2*, and *dopachrome conversion enzyme (DCE/yellow*) are involved in the darkening of eggs [12, 14–17]. However, the detailed mechanism of egg melanization in *F. taiwana* is not as well understood as that of mosquito eggs.

Therefore, to study *F. taiwana* egg melanization, we conducted RNA sequencing (RNA-seq) of pink (NaCl treated) and black (control) eggs of *F. taiwana*, and then de novo assembled and annotated the first reference transcriptome for this biting midge. We chose NaCl because it is a common and cheap reagent and offers an entry into studying *F. taiwana* melanization. Additionally, NaCl can sometimes create similar environmental conditions as the salts present in common fertilizer, so NaCl can serve as an economical proxy for some aspects of fertilizers in initial studies. We further explored the differential gene expression between the two egg sample types to better understand the molecular events associated with osmotic stress and melanization. A better understanding of the egg stage may contribute to novel strategies for *F. taiwana* control.

## Results

### *F. taiwana* transcriptome assembly and annotation

To study *F. taiwana* gene expression differences between black (control, Fig. 1A) and pink (NaCl treated; Fig. 1B) embryos, we sequenced RNA from two biological replicates of each sample type, generating 12 to 41 million raw paired-end reads per sample (Sup. Table 1 in Additional file 1). Because there is no genome sequence available yet for this species, we first produced a de novo transcriptome assembly. After Trinity assembly, data filtering, and duplicate removal, we obtained 87,415 non-redundant transcripts (Ft-nr) with an N50 of 1099 (Sup. Table 1). Of these, 26,247 (30%) transcripts were predicted to have long open reading frames (ORFs, “long” defined here as ≥300 nt). The remaining transcripts presumably consist of untranslated regions, non-coding RNAs, small peptide encoding sequences, and incomplete mRNAs.

As an initial assessment of the assembly, we examined the representation of Benchmarking Universal Single-Copy Orthologs (BUSCO) found in Ft-nr. This analysis revealed complete single or duplicate copies for 63.9 and 52.4% of the orthologs at the *Insecta* and *Diptera* taxa levels, respectively. Inclusion of the fragmented orthologs increased the corresponding coverages to 85.1 and 69.1% (Sup. Fig. 1 in Additional file 1). Saturation analysis of each RNA-seq dataset indicated that the two black (control) embryo samples did not reach saturation (Sup. Fig. 1), so these BUSCO coverage values could reflect incompleteness of the sequenced samples. Nevertheless, because only 1 life stage was examined, we consider the Ft-nr transcriptome quality and coverage to be reasonable.

Next, we annotated all Ft-nr transcripts using three approaches (annotations are in Additional files 2, 3, 4 and 5). First, functional prediction software revealed that 17.5% (15,270) of the transcripts had at least one predicted functional domain with a pfam annotation (Sup. Table 2 and Additional file 2). Second, we used blastx to compare all Ft-nr transcripts to five protein datasets. This analysis revealed that 23.4% (20,493) of the transcripts had similarity to a protein in the manually curated Swiss-Prot database and 36.9% (32,214) had similarity to that in the larger Uniref50 database (Additional file 3). We also observed comparable percentages of transcripts with putative homologs in the *Drosophila* (26.8%, 23,426), Diptera (27.4%, 23,990), and Culicidae (26.5%, 23,146) ortholog datasets (Sup. Table 2 and Additional files 4 and 5). Third, we conducted GO (Gene Ontology) and KEGG (Kyoto Encyclopedia of Genes and Genomes) Pathway analyses and found that we could annotate 25.0% (21,886) and 19% (16,625) of the transcripts with GO or KEGG terms, respectively. When we restricted our analysis to the 26,247 long ORF encoding transcripts, annotation rates were higher (Swiss-Prot, 52.2%; GO, 58.1%), as expected.

### Differentially expressed genes (DEGs)

We next conducted differential gene expression analysis by mapping the RNA-seq reads to the whole transcriptome using HiSat2 and then calling DEGs with DESeq2. This analysis revealed 6.7% (5898) of all the transcripts (and corresponding to 9.2% of all long ORFs) had log2 fold change ≥1 (i.e., 2-fold difference) between the black and the pink eggs (Sup. Fig. 2A in Additional file 1). The number of DEGs with greater expression in black eggs (88.3%, 5209 transcripts) was more that in pink eggs (11.7%, 689 transcripts; Sup. Fig. 2A).

### Principal component analysis (PCA)

To provide an overview of the gene expression data, we conducted a PCA using the whole transcriptome (Sup. Fig. 2B). The first and second principal components (PC1 and PC2) could explain 58.5 and 25.2% of the DEG variance, respectively. PC1 clearly separated the two sample types; however, PC2 was not clearly correlated with known sample properties (sampling date, sequencing run date, etc.). This result indicates that there were obvious differences in the transcriptome of the two sample conditions, consistent with the DEG analysis above.

### Gene ontology gene set enrichment analysis (GSEA) on PCA loading values

Of the 5898 DEGs, 2030 (34.4%) were annotated with GO assignments (Sup. Table 2). For these DEGs, we next conducted GSEAs using all 5898 DEGs with log2fold change ≥1 as well as the separated black (*n* = 5209) and pink (*n* = 689) up-regulated gene lists (Additional Files 6 and 7). In general, the enriched GO terms of the separated gene lists were non-overlapping subsets of that of the combined list, and thus, provide some insight into the differences between the black and pink egg sample types. For example, the black up-regulated gene set included lipid transport, axis specification, gland development, and nucleoside metabolism terms, while the pink up-regulated gene set included dopamine, pigmentation, general metabolic process, and regulation terms. We also conducted a GSEA using all of the 87,415 transcripts and using each transcript’s PC1 loading values (which separates black from pink eggs) to provide the ranking basis for the GO GSEA (Additional File 8). Because the PC1-based GSEA is not a simple fold cutoff but weighs the PC1 loading values, it should be more comprehensive, and thus we focused on this analysis.

**Fig. 2:**
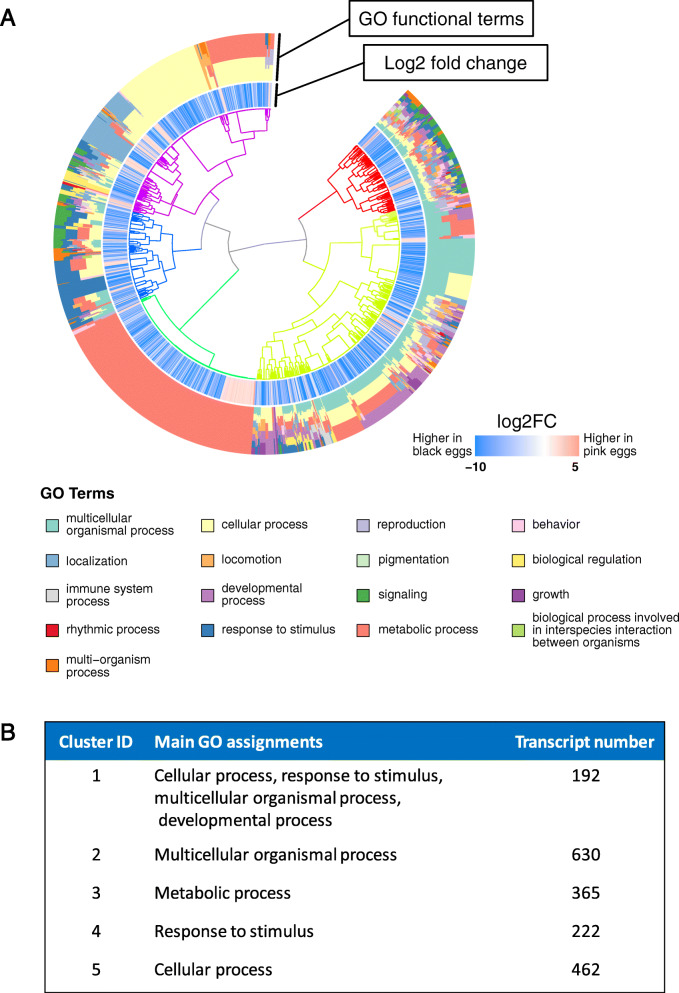
Hierarchical clustering of significantly differentially expressed transcripts with Biological Process GO Terms in *F. taiwana* eggs. **A** Clustering of 1589 DEGs into 5 main clusters (colored branches) based on the distance of the PC1 loading values (Sup. Fig. 2B) and GO assignments of each transcript. **B** Highly represented GO terms associated with each cluster. GO terms are listed if there are at least 80% of such transcripts in the cluster

We were primarily interested in the biological differences between the black and pink eggs, thus we focused only on the genes with Biological Process GO term annotations; all GSEA results including those for the molecular function and cellular compartment are in Additional files 6, 7 and 8. To permit easier understanding of the GSEA results, we hierarchically clustered the GO enriched DEGs based on the distance of the PC1 loading values and GO assignments of each transcript. A dendrogram plot shows that these 2030 DEGs, which represent 17 “level 3” Biological Process GO terms, could be separated into 5 clusters (Fig. 2). The same GO assignments were often found in multiple clusters. The GO assignments found in ≥80% of the transcripts (“highly represented”) within each group are highlighted in Fig. 2B.

**Fig. 3:**
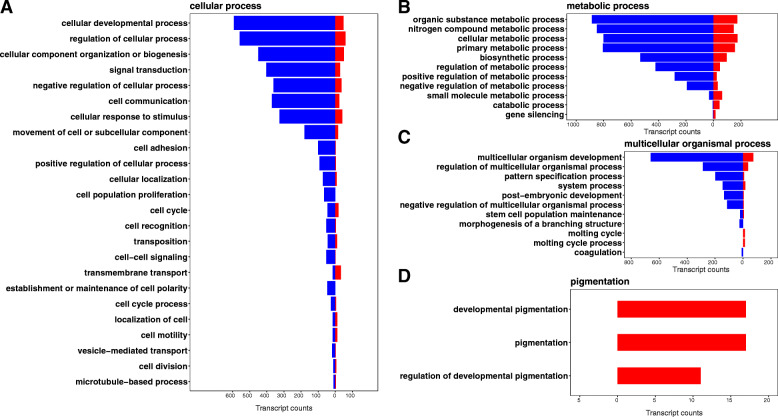
Functional distribution of the sub-categories for the highly represented and pigmentation GO categories for the DEGs between black and pink eggs. The distribution of the “next level” GO terms, separated by direction of gene expression, are shown for **A** cellular process, **B** metabolic process, **C** multicellular organismal process, and **D** pigmentation

To obtain more insight into the biological differences between the black and pink eggs, we examined the more specific “next-level” terms for the 3 highly represented GO categories (cellular process, metabolic process, and multicellular organismal process) with the most assigned transcripts. Note, in this analysis, different GO terms may have overlapping genes. Next-level GO terms were also split by whether expression was higher in black or pink eggs. For cellular process, the enriched terms were associated with cellular developmental process, cellular response to stimulus, signal transduction, cell communication, cell cycle process, and regulation of cellular process (Fig. 3A). For metabolic process, the enriched terms were associated with organic substance, nitrogen compound, small molecule and biosynthetic process, and their regulation (Fig. 3B). For multicellular organismal process, the enriched terms were associated with multicellular organism development, post-embryonic development, and regulation of multicellular organismal process (Fig. 3C). In all three cases the vast majority of the genes were more highly expressed in the black eggs.

Finally, the black and pink sample types clearly differ by pigmentation and this analysis also revealed an enrichment for the pigmentation GO term (Fig. 3D). Pigmentation sub-terms included developmental pigmentation and negative regulation of developmental pigmentation, which is critically important in the melanization pathway. However, the majority of pigmentation GO term were surprisingly up-regulated in NaCl-treated pink eggs (Fig. 3D).

### KEGG pathway analysis

We also conducted a GSEA, as above, for the DEGs with KEGG assignments (26.9%, 2030 transcripts, Table 1). This analysis revealed 18 significantly enriched KEGG pathways in 2 main categories (metabolism and environmental information processing) and a few others (Table 1, Sup. Table 3 in Additional file 1).

**Table 1:**
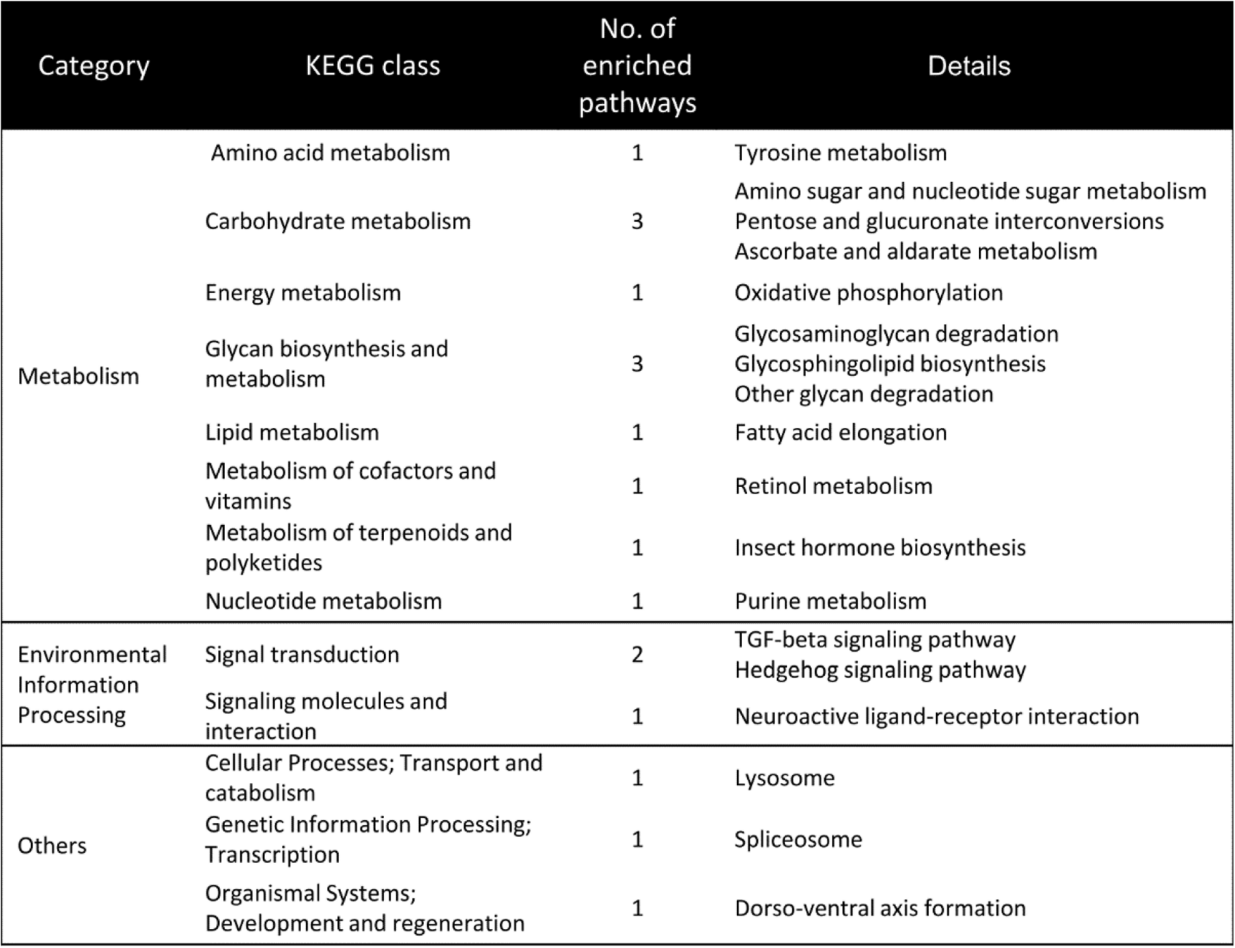
Gene Ontology gene set enrichment analysis (GSEA) for 1589 DEGs with KEGG assignment

In the metabolism category, there were 12 enriched pathways with 50% (*n* = 6) associated with carbohydrate and glycan metabolism. This may indicate that carbohydrate metabolism in eggs was dramatically changed under salt stress [18]. To a lesser extent, gene expression for other nutrition metabolism categories were altered, including lipid, amino acid, and insect hormone biosynthesis. Interestingly, retinol metabolism was enriched, as well, which is associated with light signal processing. In the environmental information processing category, 3 pathways were enriched, including the TGF-beta and Hedgehog signaling pathways, which are associated with cell proliferation, development, and differentiation.

### The melanization and osmotic stress pathway genes are differentially expressed

**Fig. 4:**
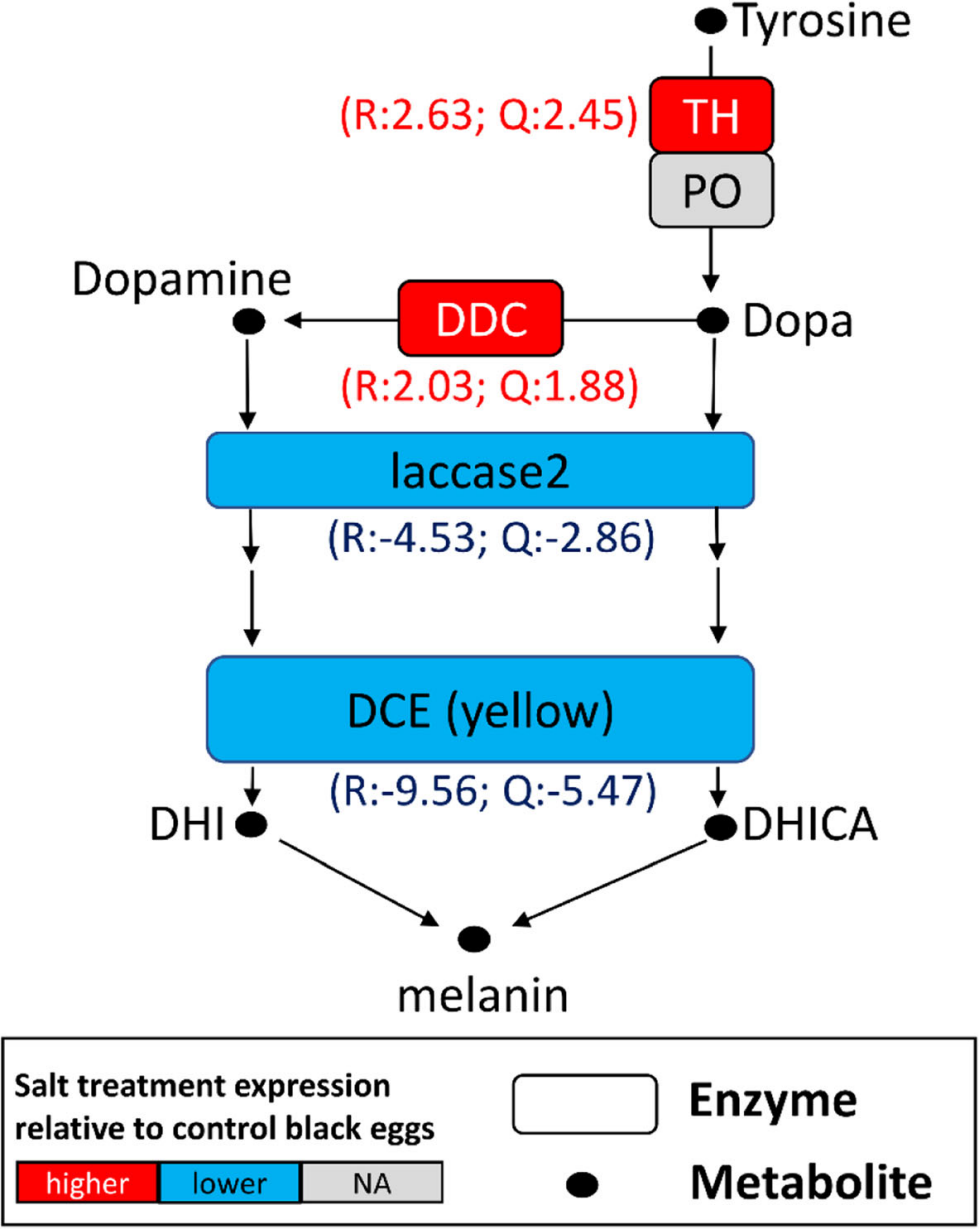
Expression patterns of genes in the melanin synthesis pathways upon salt treatment in *F. taiwana* eggs. The final two genes (*laccase2* and *DCE*) in the melanin synthesis pathway are down-regulated in NaCl-treated eggs, likely explaining why such eggs remain pink (unmelanized). R: RNA-seq, Q: qRT-PCR, both are means of log_2_ fold difference between black and pink eggs. TH: *tyrosine 3-monooxygenase*; PO: *phenoloxidase*; DDC: *Dopa decarboxylase*; DCE (yellow): *dopachrome conversion enzyme*. NA, not significant. PO has multiple isoforms and with both up- and down-regulated isoforms, so its regulation is denoted as NA. Transcript IDs are in Additional file 9

An obvious phenotypic difference between black and pink eggs is that pink eggs are un-melanized. There are 5 enzymes important for melanin synthesis as shown in Fig. 4 [19]. We hypothesized that the pink embryos would have lower expression of some or all of the genes encoding these enzymes relative to the control black eggs. Our RNA-seq data indicated that, indeed, the expression levels of the two terminal genes in the pathway, *laccase2* (− 4.53 log2FC) and *DCE/yellow* (− 9.56 log2FC), matched the expectation. However, *TH* (2.63 log2FC) and *DDC* (2.03 log2FC), which both function earlier in the pathway, were more highly expressed in pink eggs. The other gene (PO) was more complicated with both highly and lowly expressed isoforms.

NaCl treatment is a type of osmotic stress. We examined the genes associated with osmotic stress and found the up-regulation of three key genes in the NaCl-treated (pink) samples (Sup. Fig. 4A). Notably the downstream gene *duox* (*dual oxidase*) is up-regulated 2.15 fold (log_2_FC). The Duox enzyme generates H_2_O_2_ and may indicate a link with the Dopa synthesis pathway, which itself is part of the melanin synthesis pathway (Sup. Fig. 4B).

### qRT-PCR validation and *laccase2* inhibition test

We conducted qRT-PCR on independent biological samples, isolated with a slightly different NaCl concentration and feeding protocol, to confirm the RNA-seq results for 7 key genes associated with melanization or stress. Of these, 4 genes (*GST, TH, DDC,* and *DCE/yellow*) were differentially expressed and matched the direction of the RNA-seq results; another (*laccase2*) was in the same direction (significant in qRT-PCR but not significant in RNA-seq; Fig. 4, Sup. Fig. 5A). The expression of the gene *p38(MAPK)* was concordant with the RNA-seq results, with neither expression being differentially expressed. The expression of the *duox* gene was not differentially expressed and did not match the RNA-seq results (greater expression in black eggs). Overall, there was as strong, positive correlation between the RNA-Seq and qRT-PCR data for these 7 genes (Sup. Fig. 5B, *R* = 0.91, *P* = 0.0044). The lack of coherence for *duox* could be due to the different feeding protocols and/or due to the limited sample size (only 2 biological replicates for RNA-seq).

**Fig. 5:**
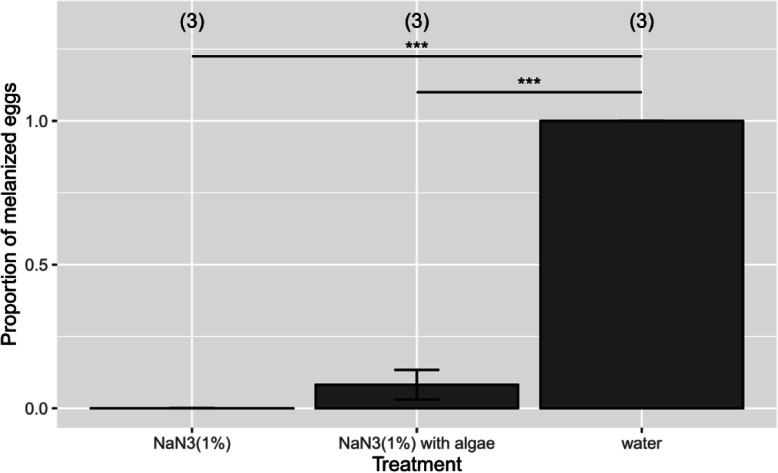
Addition of sodium azide (NaN_3_) inhibits egg melanization in *F. taiwana*. Eggs were laid by female adults onto agar containing 1% NaN_3_, agar containing 1% NaN_3_ with algae, or a tissue roll on water (control). Egg color was scored after 1 day as melanized (black) or unmelanized (pink). Sample sizes (biological replicates) are indicated in parentheses. ***, *P* < 0.001; chi-squared test

Previously studies have shown that NaN_3_ (sodium azide) is a specific inhibitor of Laccase in vitro [20]. Thus, we hypothesized that the addition of NaN_3_ (1%) into agar could inhibit Laccase and prevent melanization of biting midge eggs laid on this agar. We observed that only 10.5% of the eggs melanized, i.e., most remained pink (un-melanized), when raised on 1% NaN_3_ agar while 100% of the control eggs melanized normally (Fig. 5). Although indirect effects of NaN_3_ cannot be ruled out, this result supports the model that down-regulation of the *laccase2* (and *DCE/yellow*) gene is the mechanistic basis for the NaCl-induced inhibition of melanization.

## Discussion

Egg melanization is a normal and critical early step for *F. taiwana* development that confers protection against UV damage and desiccation. Perturbing melanization, such as by the addition of salts in the egg laying environment, prevents egg hatching, and thus, may be an entry point into controlling this pest species. In this study, we assembled the first *F. taiwana* transcriptome and then conducted a differential gene expression study comparing normal control (black) eggs with NaCl challenged (pink) eggs. These analyses yielded important information on the molecular events associated with the melanization process. In particular, our results indicate that the molecular basis for the lack of melanization in NaCl treated samples is likely due to the down-regulation of two key terminal genes in the melanin synthesis pathway.

Most insect genomes have 15–40,000 genes [21] and the only biting midge genome available is for *Culicoides sonorensis*, which has been annotated to have 15,612 genes [22]. Our Trinity assembled transcriptome contained > 87,000 genes, which seems too high. However, of these, only ~ 26,247 had ORFs with > 100 amino acids. The remainder are possibly small peptide encoding sequences, gene fragments, or UTRs that would be assembled with other genes. Some could also be regulatory RNAs or long non-coding RNAs. Another reason for the high number of transcripts is incomplete transcriptome assembly [23, 24]. Our *F. taiwana* transcriptome may contain many fragmented genes since saturation analysis showed that at least two samples (FtNB2015 and FtNB2016) were not saturated (Sup. Fig. 1). A comparison of the “long” ORF genes to the conserved insect orthologs in the BUSCO database indicated that ~ 85% of the genes were represented (Sup. Fig. 1 in Additional file 1), a reasonable value given that we sampled only one tissue. Together this suggests that the *F. taiwana* gene content might be at the higher end for insect genomes and should be resolved with a future genome sequence.

Overall, our transcriptomics results indicate differences in gene expression for 5898 of genes. The vast majority (88.3%) were more highly expressed in the viable black eggs. Many of these are general metabolism and major developmental genes, and so their reduced expression in pink eggs is consistent with failed development. Our DEG analysis does have two limitations. First, as mentioned above, two of our samples clearly did not reach saturation which may increase variance in the analysis. Second, we had only two bioreplicates per sample type, in part because obtaining samples was difficult. Despite these two shortcomings, the Pearson’s correlation coefficients of the replicate NGS datasets were high: 0.90 and 0.91 for the 2 black (control) replicates and for the 2 pink (NaCl-treated eggs) replicates, respectively. Nevertheless, some DEGs may be false positives, although at the same time we have reduced sensitivity. We used qRT-PCR to validate 7 genes (in samples treated in a slightly different way [0.1 M NaCl for qRT-PCR vs 0.25 M NaCl for RNA-seq] from those used for RNA-seq) which we found particularly relevant for melanization. We found 6 genes with concordant gene expression between qRT-PCR and the RNA-seq data and 1 that did not. So, there is high (86%) concordance despite using similar but differently processed samples, suggesting general correctness of the gene expression patterns.

Examination of the gene expression data also indicate the likely reason for the failure to melanize in NaCl treated eggs (Fig. 5). Pink eggs have significantly reduced expression levels of the two key terminal genes in the biosynthetic pathway, *laccase2* and *DCE/yellow* which sequentially catalyze the production of dopachrome or dopaminechrome and DHICA (5,6-dihydroxyindole-2-carboxylic acid) or DHI (5,6-dihydroxyindole). DHICA and DHI are further employed in the synthesis of the polymeric melanin that contribute to the pigmentation of insect eggs (Fig. 4B) [19]. In support of the role of *laccase2* in melanization, we found that embryos raised on agar containing NaN_3_, an inhibitor of Laccase [20], failed to melanize. It should be noted, however, that the lack of melanization may include additional indirect effects. Functional experiments such as with RNA interference will be required to definitely confirm the roles of these two genes.

Interestingly, several upstream genes in the melanization pathway, such as *TH* and *DDC*, were more highly expressed in the pink eggs. These two gene products produce dopa and dopamine, which also have neurotransmitter functions. Thus, one possible explanation is that their higher expression is associated with increased neural functions. A second potential explanation could be that osmotic (or other) stress normally induces melanin formation for protection, but NaCl specifically suppresses the two terminal genes. Another possibility is that the egg senses that it is not melanized and the up-regulation of upstream genes is a compensatory response in an attempt to melanize.

Salt treatment is an osmotic stress and consistent with this we found several key genes in osmotic stress up-regulated. For instance, several up-regulated genes encode *GSTs* (glutathione S-transferases), which are commonly induced for many stressors in a variety of organisms. Of note is that the *duox* (*dual oxidase*) gene in the MAP kinase stress response cascade was up-regulated. Interestingly, stress signaling through the p38 MAP kinase may also affect melanization as Duox converts oxygen to hydrogen peroxide, which is necessary for the conversion of tyrosine to Dopa, a precursor for melanin [25] (Sup. Fig. 4). p38 MAP kinase also regulates egg polarity and thus the stress response may indirectly block egg development.

Although not transmitting diseases, *F. taiwana* mainly sucks human blood in the day time [26] and thus imposes a significant impact on human health, quality of life, and economic loss. Our current studies indicated that NaCl treatment results in inviable *F. taiwana* eggs. Several fertilizers contain salts, such as urea ((NH_2_)_2_CO) and ammonium sulfate ((NH_4_)_2_SO_4_), which have been demonstrated in mosquito to inhibit development [27]. It would be interesting to test if these compounds have similar effects as NaCl toward *F. taiwana* eggs. If so, fertilizers could be incorporated into the integrated pest management (IPM) program for *F. taiwana* control. Since *F. taiwana* lay eggs in moist soil, the application of fertilizers to such soil would not only enhance the growth of crops, but also suppress the population of *F. taiwana*. This control strategy should be effective and efficient, and more importantly, eco-friendly.

More studies are clearly needed to effectively improve management efficiency of this pest. Our gene expression studies will hopefully provide a springboard for follow up studies that may lead to additional novel targets for *F. taiwana* control.

## Conclusion

In this study, the first egg transcriptome for a biting midge, *F. taiwana,* was annotated. Among the total gene set, 30% of the transcripts were predicted to have long ORFs and 17.5% have at least one predicted functional domain. A comparison between the black (control) and pink (NaCl treated) eggs revealed 5898 DEGs (40.9% of the transcripts with long ORFs) with log2 fold change ≥1 (i.e., 2-fold difference). As expected, most (88.32%) of these DEGs were down-regulated in un-melanized eggs. Despite the limited sample size, our results indicated that the two key terminal genes for melanin biosynthesis, *laccase2* and *DCE/yellow*, were significantly down-regulated in pink eggs, providing a likely mechanistic explanation for their lack of melanization. These results will be useful for further exploring the mechanism of NaCl-induced inhibition of melanization in *F. taiwana* eggs.

## Methods

### Samples

All *F. taiwana* used in this study were collected from Taichung City (GPS 24.171467, 120.751336), where endemism is highest in Taiwan, and then raised in the lab [28]. For general maintenance, *F. taiwana* were reared at room temperature in a 20 × 20 × 30 (cm^3^) transparent acrylic boxes with water and 10% (w/v) honey solution under 10 h light: 14 h dark daily cycles. To have enough RNA for sequencing, we estimated that ~ 4000 eggs were needed for each of the control and salt treatment samples. This required multiple batches of egg collections, which were obtained over an approximately one-year period as follows. In each batch, ~ 100 adult females of *F. taiwana* were blood fed 1–2 h ad libitum with blood from human volunteers (an arm was placed in the box). Three days after blood feeding, half of the *F. taiwana* females were allowed to lay eggs on 0.2% (w/v) agar plates without NaCl (controls). For the salt treatment group, the remaining females laid eggs on agar plates with 0.25 M NaCl. Because fewer eggs were laid in the salt treatment, additional egg collection batches were needed for this sample type. After 24 h, the eggs (usually 100–200) were collected into 100 μl of the Trizol reagent (Invitrogen, Carlsbad, CA, USA). Multiple batches of the same sample type were pooled together in the same tube and then extra Trizol added to a final volume of 500 μl; several such tubes were obtained for each sample type until ~ 4000 eggs were obtained. Samples were stored at -70 °C between batches. The entire collection was repeated once to obtain two biological replicates.

For the qRT-PCR validation experiments, we changed to feeding the females with an artificial blood substitute, which had been developed in the meantime [28]. Additionally, knowing that the salt treatment yielded fewer eggs, for each batch the number of adult females for the salt treatment relative to the controls was doubled. Otherwise, egg collections were done as above. Eight to nine biological replicates were tested for each gene.

### RNA extraction and sequencing

To extract RNA using the Trizol reagent, each tube of cryopreserved eggs (above) was vortexed until the egg lysate was homogeneous and clear. Afterward, 0.1 ml chloroform was added per 0.5 ml, the mixture vortexed for 3 min, and then the aqueous and organic phases separated by centrifugation for 10 min at 12,000 *g* at 4 °C (Kubota 1300). The upper aqueous phase was then precipitated with 0.5 ml isopropanol, washed with 0.5 ml 70% ethanol, dried, and finally resuspended in 30 μl water. The quantity and quality of each RNA sample was determined using a Qubit® 2.0 Fluorometer (ThermoFisher Scientific, Q32866) and a NanoVue Plus spectrophotometer (GE Healthcare Bio-Science AB, Uppsala, Sweden). In all four cases, the A260:280 ratios were between 1.8 and 2.0 and the A260:230 ratios were between 2.0 and 2.4.

Total RNA samples were adjusted to 100 ng/μl and prepared for sequencing using the TruSeq® Stranded mRNA library prep protocol. RNA sequencing (RNA-seq) was done at the Next Generation Sequencing Core Facility (Center for Biotechnology, National Taiwan University, Taipei, Taiwan) on the Illumina HiScanSQ (2x100 bp, 2015 and 2016) or Illumina NextSeq500 (2x150 bp, 2017) platforms (Table 1).

### De novo transcriptome assembly, BUSCO analysis, coding region prediction, and functional annotation

Raw reads were processed by trimming the adaptor sequence, retaining only bases with quality score > 14, and then removing those with < 25 nt remaining using Trimmomatic (v.0.39) [29]. After, FastQC [30] was used to confirm the quality of the trimmed reads; all reads were 25–141 nt in length. Additionally, a saturation analysis was conducted on each of the four RNA-seq dataset (Sup. Fig. 3).

A de novo transcriptome assembly was generated using Trinity from the combined post-processed reads from egg samples, using the default parameters [31]. Next, contaminating transcripts matching the kmer profiles of bacteria, fungi, virus or archaea were removed using Kraken2 [32]. However, we re-included the *laccase2* gene (ID: TRINITY_DN68680_c0_g1_i1), which was inappropriately classified as a contaminant by Kraken2, because of its central role in melanin synthesis. Transcripts were also removed if blast homology searches against all algae mRNAs from NCBI and the genomes of *Arabidopsis thaliana, Caenorhabditis elegans, Drosophila melanogaster, Danio rerio, Homo sapiens, Mus musculus,* Phage lambda, *Persea americana, Rattus norvegicus, Saccharomyces cerevisiae,* and *Xenopus laevis* yielded strong hits (Evalue < 0.01 and query coverage > 70%) but did not include *D. melanogaster*. We included plant sequences (algae, *A. thaliana*, and the avocado plant, *P. americana*) because biting midge larvae sometimes feed on algae and preliminary analyses indicated some contamination from avocado. After, short sequences (< 200 bp) and sequences with very low abundance of coverage (average less than 2 reads after deduplication) were removed on the presumption that these genes were unreliable. Finally, the rRNAs genes were identified by blast (Additional file 10) and removed from subsequent the analysis. To generate a non-redundant (Ft-nr) gene set, CD-HIT-EST (v.4.8.1) was used to cluster transcripts with ≥95% nucleotide identity [33]. Then BUSCO (v.4.0.0) was used to assess the completeness of Ft-nr [34].

For functional annotation, coding regions were first predicted using TransDecoder (v5.0.2) [35]. Then, following the suggestions of the Trinotate script [31], only the open reading frames predicted to encode proteins with ≥100 amino acids were used for function prediction and motif finding. Hmmscan (v.3.3) [36], SignalP (v.5.0) [37], and TmHMM (v.2.0) [38] were used to annotate putative pfam motifs [39], signal peptides, and transmembrane proteins, respectively. Additionally, a BLASTn search (sequence identity ≥75% and E-value ≤10e-3) against the TREP database (trep-db_nr_Rel-19.fasta) [40] was used to identify likely transposable elements (TEs). Bioinformatics command line code are in the Additional file 11.

### Homology prediction, gene ontology (GO) term, and KEGG pathway assignment

Homology predictions for all Ft-nr transcripts were determined by separate DIAMOND blastx (v0.9.29) [41] similarity searches against the UniProt (downloaded on 2019/12/16) and UniRef50 databases (v.3.1, downloaded on 2019/12/20) [42]. In addition, all Ft-nr transcripts were compared against the *D. melanogaster* proteome (dmel-all-translation-r6.30, downloaded on 2019/12/16 from Flybase) [42] and the predicted orthologs from the *Diptera* and *Culicidae* taxa levels in OrthoDB (v.10) [43]. All results were loaded into a Trinotate SQLite Database which was then used to assign eggnog [44], GO (Gene Ontology) [45] and KEGG pathway terms [46].

### Differential expression analysis

Hisat2 (v2.1.0) [47] was used to map trimmed reads of each sample to Ft-nr, and then mapped reads were sorted by samtools (v1.10) [48]. StringTie (v.2.0.6) [49] was used to estimate the abundance of each transcript and build the count matrix for each sample. To account for batch effects, the RNA-seq protocol information was included in the model to normalize the counts matrix then analyzed for differential gene expression using the DESeq2 package (v1.26.0) [50] from Bioconductor (v.3.11) [51] on the R platform (v.3.6.3) [52].

### Principal component analysis (PCA)

The StringTie expression count output data were log2 transformed by DESeq2::rlogTransformation (blind = TRUE) and then imported into stats::prcomp (scale = TRUE) for PCA. The PCA loading values for principle component 1 (PC1) and PC2 were extracted for the next analysis.

### Gene ontology gene set enrichment analysis (GSEA) on PC loading values

GO assignments were updated to the latest synonymous terms by filtering obsolete terms following the GO.db package (v 3.11.1) [53]. After, GSEA was performed using the GSEA function from the clusterProfiler package [54] to produce 3 enriched GO lists based on the gene expression level (log2 fold change [log2FC]) or the loading values for PC1 or PC2. The Biological Process GO terms for the enriched differentially expressed genes (DEGs) by both log2FC and PC1 were then used to calculate the distances of each transcript using stats::dist (method = euclidean) and for clustering using stats::hclust (method = ward. D2). The GOplot package (v.1.0.2) was used for plotting [55].

### KEGG pathway analysis

All KEGG pathway assignments were pruned to contain only the pathways belonging to *D. melanogaster*, *Anopheles gambiae*, *Aedes aegypti*, *Aedes albopictus* or *Culex quinquefasciatus* using the KEGGREST package (v. 1.28) [56]. Significant enrichment of the KEGG pathways was determined as above (for GO GSEA) based on the log2FC gene expression level or the loading values for PC1 or PC2. Additionally, a hypergeometric enrichment test was conducted on the list of differentially expressed genes with post-pruned KEGG pathway assignments with clusterProfiler::enricher [54]. All significantly enriched KEGG pathways were then grouped into more general pathway categories using the KEGGREST package.

### Quantitative real time PCR (qRT-PCR)

qRT-PCR was conducted following the SensiFAST™ SYBR® Hi-ROX Kit with the following cycle conditions: 2 min 95 °C initial denaturation followed by 40 cycles of 2-step cycling (5 s 95 °C denaturation and 30 s 60 °C annealing and extension step). The reactions were run on an Applied Biosystems™ StepOnePlus™ Real-Time PCR System. For each gene, 8 to 9 biological replicates were tested, and each biological replicate consisted of 3 technical replicates. The *tubulin* gene was used as the internal control and all gene expression values were scaled relative to it. The F test was used to test if the sample variances were normally distributed (stat::var.test (alternative = “two.sided”)), and the unpaired two-tailed t-test (t.test (paired = F, var.equal = F/T [depending on F test])) was used to test for differences in the relative gene expression values (2^(−ΔΔCt)). Standard error (FSA::se()) [57] was also calculated. Statistical analyses were done in R (v.3.6.3). Primer information is in Sup. Table 4 in Additional file 1.

### Inhibition of melanization with NaN_3_

In each biological replicate, ~ 50 *F. taiwana* adult females were blood-fed with an artificial blood substitute and maintained in 20 × 20 × 20 (cm^3^) transparent acrylic box for 2 days. Three types of media were prepared for egg laying: 2% agar plate plus 1% NaN_3_ (i.e., 0.15 M), 2% agar plate plus 1% NaN_3_ with algae solution, and a wet tissue paper roll placed in a water bottle. Subsequently, the three media were put into the same box. Adult females were added to the box in the morning (~ 9:30 am). Eggs were collected after 24 h and scored for melanization. Three biological replicates were tested.

## Supplementary Information

**Additional file 1.**

**Additional file 2.**

**Additional file 3.**

**Additional file 4.**

**Additional file 5.**

**Additional file 6.**

**Additional file 7.**

**Additional file 8.**

**Additional file 9.**

**Additional file 10.**

**Additional file 11.**

## Data Availability

RNA-seq data have been deposited in the NCBI Short Read Archive (SRA) as accession numbers: SRR13426333 to SRR13426336. This study is BioProject PRJNA691747 (https://www.ncbi.nlm.nih.gov/bioproject/PRJNA691747). The assembled transcriptome has been deposited in the NCBI Transcriptome Shotgun Assembly (TSA) as accession number: GIYU01000000.
