# Supplementary material for: Transcriptome profiling reveals the developmental regulation of NaCl-treated Forcipomyia taiwana eggs
Source: BMC Genomics. 2021 Nov 3;22:792. doi: 10.1186/s12864-021-08096-x (PMC8567638; doi:10.1186/s12864-021-08096-x)
Supplement: Supplementary file 1 — Additional file 1. [file 12864_2021_8096_MOESM1_ESM.pdf]

Supplemental information 1

Transcriptome profiling reveals the developmental regulation of NaCl-treated *Forcipomyia taiwana* eggs

Mu-En Chen[1,2], Mong-Hsun Tsai[3,4,5], Hsiang-Ting Huang[1], Ching-Chu Tsai[1] , Mei-Ju Chen[4], Da-Syuan Yang[1], Teng-Zhi Yang[1], John Wang[2]*, Rong-Nan Huang[1]*

[1] Department of Entomology and Research Center for Plant Medicine, College of Bioresources and Agriculture, National Taiwan University, Taipei 10617, Taiwan

[2] Biodiversity Research Center, Academia Sinica, Taipei 11529, Taiwan

[3] Institute of Biotechnology, College of Bioresources and Agriculture, National Taiwan University, Taipei 10617, Taiwan

[4] Centers for Genomics and Precision Medicine, National Taiwan University, Taipei 10617, Taiwan

[5] Agricultural Biotechnology Research Center, Academia Sinica Taipei 11529, Taiwan

* Author for correspondence: Rong-Nan Huang (rongent@ntu.edu.tw) and John Wang (johnwang@gate.sinica.edu.tw)

Contents of all additional files

Additional file 1:

This file. Contains Supplementary Figures 1 to 5 and Tables 1 to 4.

Additional file 2:

Table of the standard Trinotate annotation information for each transcript. gene_id and transcript_id: IDs from Trinity output; prot_id and prot_coords: ORF ID, position, and strand orientation from TransDecoder output; Pfam: Annotation information from Pfam database via hmmscan; SignalP: Annotation data from signalp software; TmHMM: Annotation data from tmhmm software; sprot_Top_BLASTX_hit and sprot_Top_BLASTP_hit: Annotation data from Swiss-Prot database; eggnog: Annotation data from eggNOG database via Trinotate; Kegg: Annotation data from Kegg database via Trinotate; gene_ontology_BL0041STX, gene_ontology_BLASTP, and gene_ontology_Pfam: Annotation data from the Gene Ontology (GO) knowledgebase via blastx, blastp and hmmscan, respectively.

Additional file 3:

Table of annotation based on the Uniref50 and TREP databases for each transcript. gene_id and transcript_id: IDs from Trinity output; trep-db_nr_Rel-19_BLASTn: Annotation data from TREP database via blastn; uniref50_BLASTX: Annotation data from UniRef50 database for proteins sequences with at least 50% sequence identity to and 80% overlap with the longest sequence in the cluster (Suzek et al., 2014) via blastx.

Additional file 4:

Table of annotation based on the Flybase database for each transcript. gene_id and transcript_id: IDs from Trinity output; diamond_dmel-all-translation-r6.30.fasta_BLASTX: Annotation data from Flybase annotated Drosophila melanogaster proteins via blastx.

Additional file 5:

Table of annotation based on the OrthoDB database and restricted to the Diptera and Culicidae clades for each transcript. gene_id and transcript_id: IDs from Trinity output; OrthoDB.Diptera_BLASTX and OrthoDB.Culicidae_BLASTX: Annotation data from orthoDB database limited to orthologous gene sequences in the Diptera and Culicidae clades via blastx.

Additional file 6:

Table of full results of Gene Set Enrichment Analysis (GSEA) based on the $\log_2$ fold

change value for the differentially expressed genes with GO assignments. Genes were included if their absolute log_2 fold change was greater than or equal to 1. This list contains 5,898 transcripts. Enrichment scores are based on a weighted Kolmogorov-Smirnov-like statistic implemented in the clusterProfiler package (Yu et al., 2012).

Additional file 7:
Tables of Gene Set Enrichment Analysis (GSEA) for GO terms conducted separately for the genes up-regulated in black or pink eggs. Genes were included if their absolute log_2 fold change was greater than or equal to 1. This list contains 5209 up-regulated transcripts in black eggs and 689 up-regulated transcripts in pink eggs. Enrichment scores are based on a weighted Kolmogorov-Smirnov-like statistic implemented in the clusterProfiler package (Yu et al., 2012).

Additional file 8:
Table of full results of Gene Set Enrichment Analysis (GSEA) based on the principal components analysis loading values for the differentially expressed genes with GO assignments. Genes were included if their absolute log_2 fold change was greater than or equal to 1. This list contains 87,415 transcripts. Enrichment scores are based on a weighted Kolmogorov-Smirnov-like statistic implemented in the clusterProfiler package (Yu et al., 2012).

Additional file 9
Table of the gene information in Fig. 4 with the log2fold changes and the annotation data.

Additional file 10
Table of all rRNAs identified by blast against 16S, ITS, LSU and rRNA of *Drosophila melanogaster* from NCBI website.

Additional file 11:
List of all command lines used in the bioinformatics analyses.

List of supplementary figures and tables.

Sup Fig. 1 Saturation analysis of the RNA-seq datasets
Sup Fig. 2 Overview of the Benchmarking Universal Single-Copy Orthologs (BUSCO) analysis
Sup Fig. 3 Overview of the gene expression differences between black (normal, control) and pink (NaCl treated) *F. taiwana* eggs.
Sup Fig. 4 Expression patterns of genes in the osmotic stress signaling and melanin synthesis pathways upon salt treatment in *F. taiwana* eggs.
Sup Fig. 5 qRT-PCR validation of 7 melanin formation genes identified as DEGs by RNA-seq in *F. taiwana* eggs.
Sup Table 1 Summary of RNA-seq metrics for *F. taiwana* egg transcriptomes under NaCl stress
Sup Table 2 Summary of gene annotations for the *F. taiwana* egg transcriptomes
Sup Table 3 Full results of Enrichment Analysis for the differentially expressed genes with KEGG assignments.
Sup Table 4 List of primers used for quantitative real-time PCR.

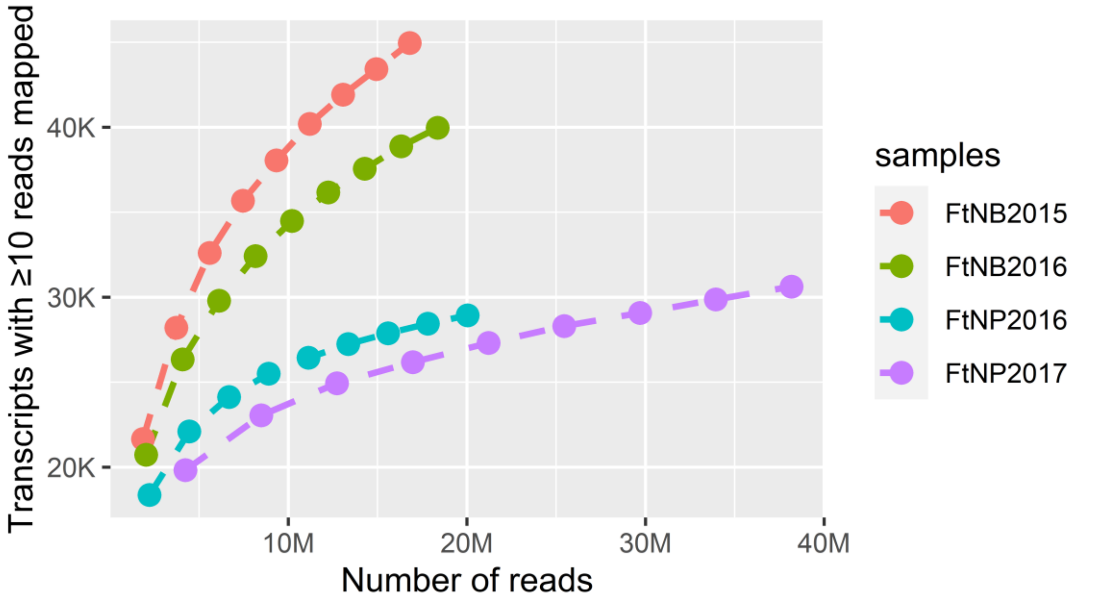

Sup Fig. 1 Saturation analysis of the RNA-seq datasets. Points are the number of *F. taiwana* non-redundant transcripts with ≥10 mapped reads at a given amount of reads sampled. The amount of reads sampled increased in 10% increments of the total for each dataset.

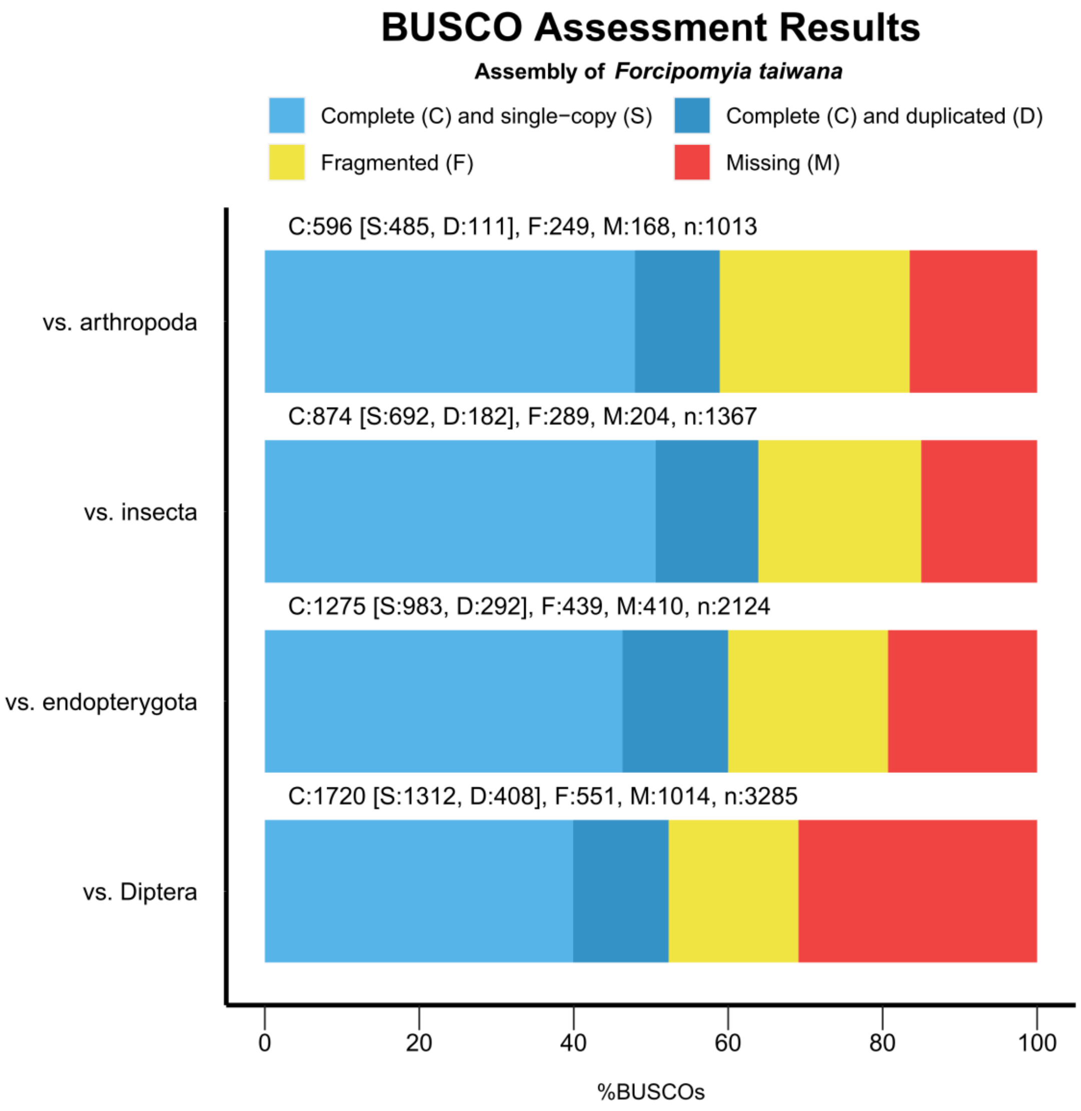

Sup Fig. 2 Overview of the Benchmarking Universal Single-Copy Orthologs (BUSCO) analysis. BUSCO genes found in Ft-nr by comparison against the *arthropoda, insecta, endopterygota*, and *Diptera* taxa. C, complete-copy; S, single-copy; D, duplicated; F, fragmented; M, missing.

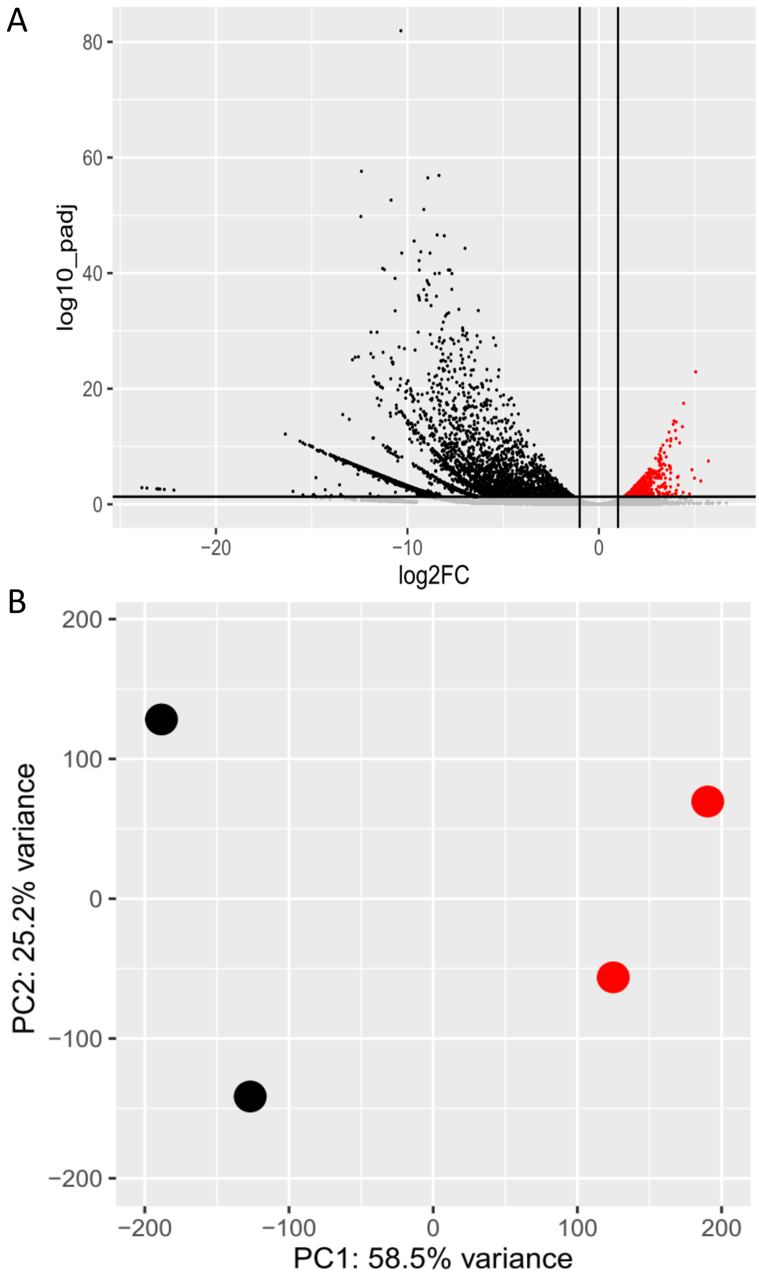

Sup Fig. 3 Overview of the gene expression differences between black (normal, control) and pink (NaCl treated) *F. taiwana* eggs. (A) Volcano plot of gene expression between black and pink *F. taiwana* eggs. There were 5209 (88.32%) higher expressed transcripts in black eggs and 689 (11.68%) higher expressed transcripts in pink eggs. (B) Principal component analysis (PCA) of the four samples of *F. taiwana* eggs (two biological replicates for each sample type) based on normalized mRNA expression levels. PC1 separates the samples by treatment.

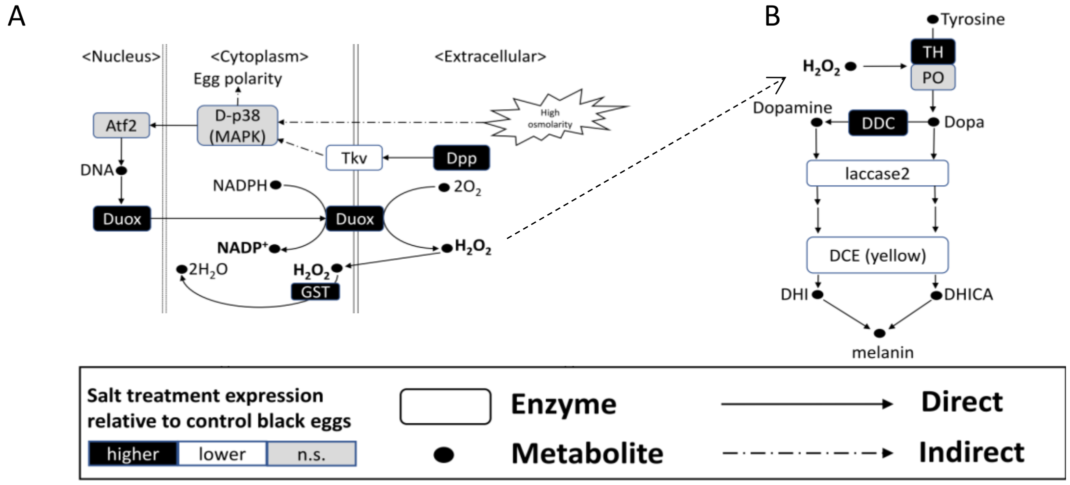

Sup Fig. 4 Expression patterns of genes in the osmotic stress signaling and melanin synthesis pathways upon salt treatment in *F. taiwana* eggs. (A) Salt treatment is an osmotic stress which could trigger the production of H_2O_2 through the MAPK signal pathway (Farnesi et al., 2017), as suggested by the up-regulation of *GST* (da Silva et al., 2019). The osmotic stress pathway is indirectly linked to the melanin synthesis pathway through H_2O_2. (B) The final two genes (*laccase2* and *DCE*) in the melanin synthesis pathway are down-regulated in salt treated eggs, likely explaining why such eggs remain pink (unmelanized). Dpp: *decapentaplegic*; Tkv: *thickveins*; D-p38: *p38c MAP kinase*; Atf2: *cyclic AMP-dependent transcription factor*; Duox: *dual oxidase*; GST: *glutathione S-transferase*; TH: *tyrosine 3-monooxygenase*; PO: *phenoloxidase*; DDC: *Dopa decarboxylase*; DCE (yellow): *dopachrome conversion enzyme*. n.s., not significant. PO has multiple isoforms and with both up- and down-regulated isoforms, so its regulation is denoted as n.s. Transcript IDs are in Additional file 9.

A.

B.

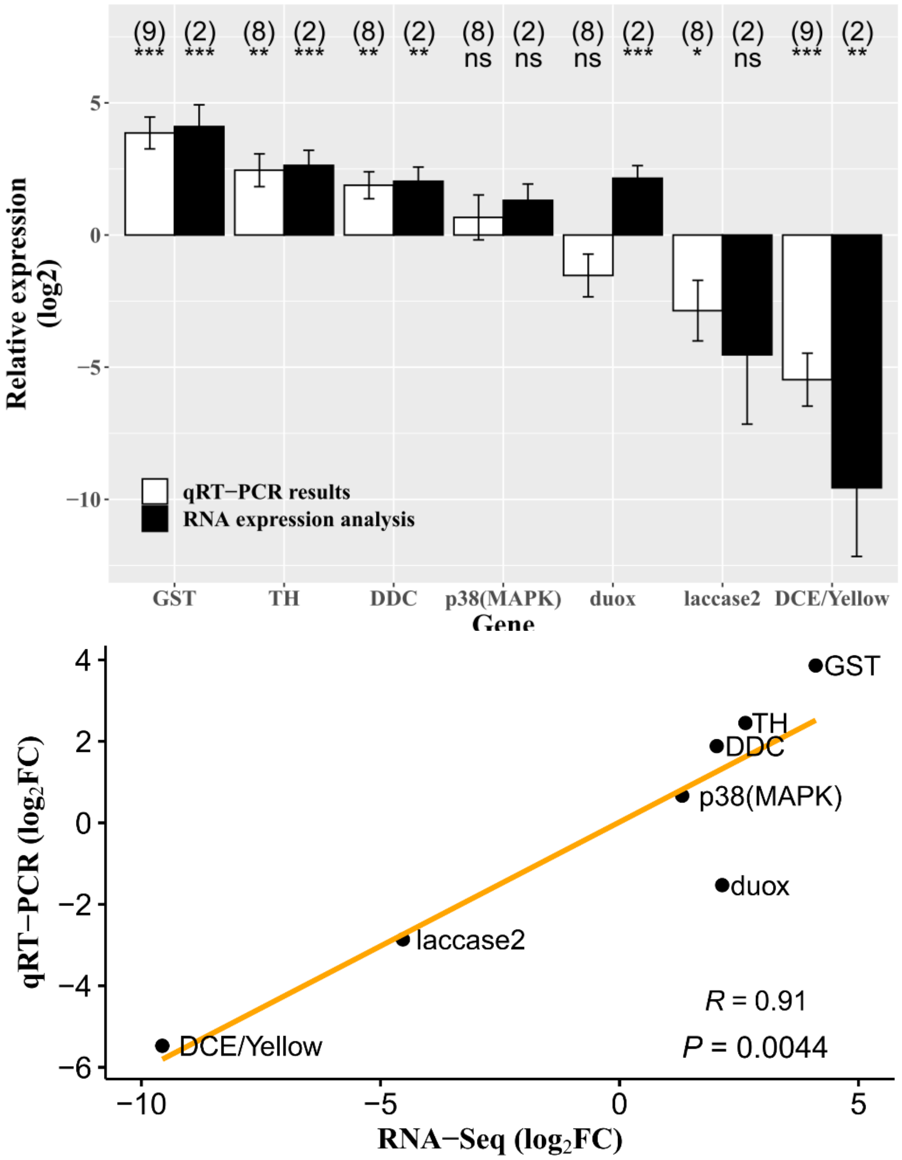

Sup Fig. 5 qRT-PCR validation of 7 melanin formation genes identified as DEGs by RNA-seq in *F. taiwana* eggs. **(A)** qRT-PCR was used to measure mRNA levels in pink eggs (treated with 0.1 M NaCl) and black eggs (untreated controls). Relative expression levels (mean±SE) are presented for both qRT-PCR (black bars) and RNA-seq (white bars) results. Sample sizes (biological replicates) are indicated in parentheses. Statistical significance values of differential expression are from the *t*-test (qRT-PCR) or DESeq statistical test (RNA-seq); ***, $P < 0.001$; **, $P < 0.01$; *, $P < 0.05$; ns, not significant. **(B)** Correlation plot of RNA expression data and qRT-PCR results. Orange line is the linear regression fit. The Pearson correlation coefficient (R) and the P-value (P) are indicated.

Sup Table 1 Summary of RNA-seq metrics for *F. taiwana* egg transcriptomes under NaCl stress

**Raw data**				
ID	Condition	Raw reads (PE*)	Trimmed reads (PE*)	Mapped reads‡
FtNB2015	control	12,166,142	12,125,464	18,665,417 (76.97%)
FtNB2016	control	NA	13,601,478	20,405,665 (75.01%)
FtNP2016	NaCl	NA	15,156,465	22,265,848 (73.45%)
FtNP2017	NaCl	41,208,806	32,307,432	42,427,208 (65.66%)

De novo assembly (by Trinity)				
	Counts	Total length	N50	%GC
Assembled Contigs	163,454	104.8 MB	1,146	46.44
Cleaned contigs	129,052	89.79MB	1,193	46.27
Clustered contigs+	87,415	60.53MB	1,099	46

* Number of paired-end reads

‡ Each read of a pair is counted separately for mapping (percent of all reads, i.e., 100 x mapped reads/(2 x total Trimmed reads))

+ After CD-HIT-EST clustering at 95% similarity

NA: Raw reads from these two samples (FtNB2016 and FtNP2016) have been lost.

Sup Table 2 Summary of gene annotations for the *F. taiwana* egg transcriptomes

Annotated contigs				
	Database	All transcripts (n=87,415)	Transcripts with ORFs ≧100 a.a. (n=26,247)	DEGs (n=5,898)
Functional domain prediction	TransDecoder (predicted ORF)	26,247 (30%)	26,247 (100%)	2,415 (40.9%)
	Pfam (functional profile)	15,270 (17.5%)	15,270 (58.2%)	1,446 (24.5%)
	SignalP (signal peptide)	2,454 (2.8%)	2,454 (9.3%)	212 (3.6%)
	TmHMM (transmembrame protein)	3,997 (4.6%)	3,997 (15.2%)	384 (6.5%)
	Trep (transposons)	4 (0.005%)	0(0%)	0 (0%)
BLAST in 10^{-3} E-value cutoff	Swiss-prot BLASTX	20,493 (23.4%)	13,714 (52.2%)	1,923 (32.6%)
	Swiss-prot BLASTP (ORF ≧ 100 a.a.)	13,346 (15.3%)	13,346 (50.8%)	1,278 (21.7%)
	Uniref50			
	Dmel-AllTranslation (drosophila gene)	32,214 (36.9%)	19,739 (75.2%)	2,927 (49.6%)
	OrthoDB_Diptera	23,426 (26.8%)	15.579 (59.4%)	2,179 (36.9%)
	OrthoDB_Culicidae	23,990 (27.4%)	15,550 (59.2%)	2,207 (37.4%)
Trinotate assignments	eggNOG (ortholog)	23,146 (26.5%)	15,284 (58.2%)	2,123 (36%)
	Gene Ontology* (gene feature such as localization, function..)	17,239 (19.7%)	11,701 (44.6%)	1,656 (28%)
	KEGG (pathway)	21,886 (25%)	15,262 (58.1%)	2,030 (34.4%)

* Combined blastx, blastp and pfam results, see Supplementary file 2.

Sup Table 3 Full results of Enrichment Analysis for the differentially expressed genes with KEGG assignments

Analysis[1]	Higher in[2]	KEGG pathway ID	KEGG Name	pvalue	p.adjust	qvalue	Count	KEGG Class
**HG**		map04320	Dorso-ventral axis formation	1.20E-07	6.72E-06	5.05E-06	23	Organismal Systems; Development and regeneration
**HG**		map00350	Tyrosine metabolism	9.93E-07	2.78E-05	2.09E-05	17	Metabolism; Amino acid metabolism
**HG**		map04350	TGF-beta signaling pathway	0.002822	0.022578	0.016976	16	Environmental Information Processing; Signal transduction
**HG**		map00520	Amino sugar and nucleotide sugar metabolism	0.004128	0.025683	0.019311	21	Metabolism; Carbohydrate metabolism
**HG**		map00062	Fatty acid elongation	0.000338	0.006305	0.004741	15	Metabolism; Lipid metabolism
**HG**		map04341	Hedgehog signaling pathway - fly	0.002467	0.022578	0.016976	13	Environmental Information Processing; Signal transduction
**HG**		map00830	Retinol metabolism	0.006151	0.034448	0.025901	12	Metabolism; Metabolism of cofactors and vitamins
**HG**		map00053	Ascorbate and aldarate metabolism	0.002472	0.022578	0.016976	12	Metabolism; Carbohydrate metabolism
**HG**		map00531	Glycosaminoglycan degradation	0.000672	0.00941	0.007075	12	Metabolism; Glycan biosynthesis and metabolism

**HG**		map00981	Insect hormone biosynthesis	0.008912	0.045368	0.034112	8	Metabolism; Metabolism of terpenoids and polyketides
**HG**		map00604	Glycosphingolipid biosynthesis - ganglio series	0.003693	0.025683	0.019311	6	Metabolism; Glycan biosynthesis and metabolism
**log2FC**	black eggs	map03040	Spliceosome	0.00361	0.045126	0.039901	22	Genetic Information Processing; Transcription
**log2FC**	black eggs	map00190	Oxidative phosphorylation	0.002597	0.045126	0.039901	20	Metabolism; Energy metabolism
**PC1**	black eggs	map01100	Metabolic pathways	0.000978	0.008154	0.00515	268	NA
**PC1**	black eggs	map04320	Dorso-ventral axis formation	0.000301	0.003759	0.002374	23	Organismal Systems; Development and regeneration
**PC1**	pink eggs	map04142	Lysosome	0.007381	0.030755	0.019424	19	Cellular Processes; Transport and catabolism
**PC1**	black eggs	map00350	Tyrosine metabolism	0.000277	0.003759	0.002374	17	Metabolism; Amino acid metabolism
**PC1**	pink eggs	map04350	TGF-beta signaling pathway	0.003286	0.020537	0.012971	16	Environmental Information Processing; Signal transduction
**PC1**	pink eggs	map00511	Other glycan degradation	0.005295	0.026473	0.016719	10	Metabolism; Glycan biosynthesis and metabolism
**PC2**		map04320	Dorso-ventral axis formation	0.00623	0.025958	0.016395	23	Organismal Systems; Development and regeneration

**PC2**	map00230	Purine metabolism	0.000227	0.002843	0.001795	22	Metabolism; Nucleotide metabolism
**PC2**	map00520	Amino sugar and nucleotide sugar metabolism	0.002141	0.013381	0.008451	21	Metabolism; Carbohydrate metabolism
**PC2**	map04080	Neuroactive ligand-receptor interaction	0.000227	0.002843	0.001795	21	Environmental Information Processing; Signaling molecules and interaction
**PC2**	map04341	Hedgehog signaling pathway - fly	0.002924	0.01462	0.009234	13	Environmental Information Processing; Signal transduction
**PC2**	map00040	Pentose and glucuronate interconversions	0.001452	0.012099	0.007642	12	Metabolism; Carbohydrate metabolism

[1] One of the following 4 analysis types
HG: Enrichment analysis based on the hypergeometric test
log2FC: Gene set enrichment analysis (GSEA) based on $\log_2$ fold change of each gene
PC1: GSEA based on the loading value of principle component 1
PC2: GSEA based on the loading value of principle component 2

[2] PC1 separates samples by egg color, so the egg type with higher expression is indicated; not relevant for other analysis types

Sup Table 4 List of primers used for quantitative real-time PCR.

Primers	Sequence (5'-3')	Length	TM(°C)
**Duox-5F**	CGAAATGATGCGGTCTCTCGTTGAAATAG	29	60.1
**Duox-5R**	CCC TTG TAC TCC TTC ATC ATG AGC TTG	27	59.7
**p38-1F**	CAC GAA TCT TAG TAA TGT GTG GAT CTC	27	56.7
**p38-1R**	TTT CTC CAG TAA ATC TAT TGC TTG TGG	27	55.2
**laccase2-3F**	CAG TGT ACG AGA TGA AGC TAA TAA AAG	27	55.2
**laccase2-3R**	TTT GCT CAC TTT TAA ACT TTA CAC TCT TTG	30	54.8
**Tubulin7_F**	CCA TTG GAG GCG GTG ATT ACT C	22	56.7
**Tubulin7_R**	GGT CTC CAA GTC GAC GAA CAC	21	56.3
**GstDrs2_F**	AGG ACT TCT ACT AAC TTC CCG G	22	54.8
**GstDrs2_R**	GTT CAG AAT CTT GAG GTT CAC GTC	24	55.7
**DDC5_F**	GAC ATC AAG TAC TCA TGG ACC G	22	54.8
**DDC5_R**	TCC ACC ATT CCC GAT TCG AAG	21	54.4
**TH5_F**	CGG GAG ATG GTT AGC TTG GG	20	55.9
**TH5_R**	AAC TGA CAG CCC TCG GTA TTG	21	54.4
**yellow3_F**	AAC CAG GTC CGT GTT CTT CAG	21	54.4
**yellow3_R**	GAT AGC TCT CCT CAA TGC CCT C	22	56.7
