# Supplementary material for: Transcriptome profiling reveals the developmental regulation of NaCl-treated Forcipomyia taiwana eggs
Source: BMC Genomics. 2021 Nov 3;22:792. doi: 10.1186/s12864-021-08096-x (PMC8567638; doi:10.1186/s12864-021-08096-x)
Supplement: Supplementary file 11 — Additional file 11. [file 12864_2021_8096_MOESM11_ESM.docx]

**Supplemental information 9**

Transcriptome profiling reveals the developmental regulation of NaCl-treated *Forcipomyia taiwana* eggs

Mu-En Chen^a,b^ , Mong-Hsun Tsai^c,d,e^, Hsiang-Ting Huang^1^, Ching-Chu Tsai^a^ , Mei-Ju Chen^d^, Da-Syuan Yang^a^, Teng-Zhi Yang^a^, John Wang^b^*, Rong-Nan Huang^a^*

^a^ Department of Entomology and Research Center for Plant Medicine, College of Bioresources and Agriculture, National Taiwan University, Taipei 10617, Taiwan

^b^ Biodiversity Research Center, Academia Sinica, Taipei 11529, Taiwan

^c^ Institute of Biotechnology, College of Bioresources and Agriculture, National Taiwan University, Taipei 10617, Taiwan

^d^ Centers for Genomics and Precision Medicine, National Taiwan University, Taipei 10617, Taiwan

^e^ Agricultural Biotechnology Research Center, Academia Sinica Taipei 11529, Taiwan

* Author for correspondence: Rong-Nan Huang (rongent@ntu.edu.tw) and John Wang (johnwang@gate.sinica.edu.tw)

**Table of Contents:**

A. Sequence processing, assembly and mapping.

1. Raw data trimming

2. De novo transcriptome assembly

3. Clustering transcripts

4. Predicting open reading frames

5. Mapping trimmed reads to Trinity assembly

6. Sorting the sam data

B. Annotation

1. BUSCO analysis of the Trinity assembly

2. Trinotate annotation

a. pfam

b. signalp(v5.0)

c. tmhmm(v2.0)

d. uniprot_sprot

e. Drosophila coding-genes

f. uniref50

g. OrthoDB.Diptera

h. OrthoDB.Culicidae

i. TREP database (v. 2019)

C. Differential expression analysis, principal component analysis and enrichment analysis.

1. Estimating the abundance and building up the count matrix

2. Differential expression analysis

3. Principal Components Analysis

4. Gene set enrichment analysis

5. Hypergeometric test

D. Other statistical analyses

1. F test

2. t test

3. Standard error

**Bioinformatics command line code**

1. **Sequence processing, assembly and mapping.**

1. Raw data trimming

Trimmomatic (v 0.39) on bash platform

java -jar /PATH/Trimmomatic-0.39/trimmomatic-0.39.jar PE \

-threads 8 \

/PATH/data_R1_001.fastq.gz \

/PATH/data_R2_001.fastq.gz \

data_forward_paired.fq.gz \

data_forward_unpaired.fq.gz \

data_reverse_paired.fq.gz \

data_reverse_unpaired.fq.gz \

ILLUMINACLIP: /PATH/Trimmomatic-0.39/adapters/TruSeq2-PE.fa:2:30:10 \

ILLUMINACLIP: /PATH/Trimmomatic-0.39/adapters/TruSeq3-PE.fa:2:30:10 \

HEADCROP:10 MINLEN:34

2. De novo transcriptome assembly

Trinity (v2.0.6) on bash platform

/PATH/Trinity \

--seqType fq \

--max_memory 130G \

--left data.fastq --right data.fastq \

--CPU 14 \

--output Trinity_v2.0.6 \

--group_pairs_distance 180 \

--SS_lib_type RF

3. Clustering transcripts

CD-HIT-est (v4.8.1-2019-0228) on bash platform

/PATH/cd-hit-est \

-c 0.95 -n 9 \

-T 24 -M 16000\

-i /PATH/Trinity.fasta -o Trinity.CDHITe

4. Predicting open reading frames

TransDecoder (v‎5.0.2) on bash platform

/PATH/TransDecoder.LongOrfs -t data

/PATH/TransDecoder.Predict -t data

5. Mapping trimmed reads to Trinity assembly

hiset2 (v2.1.0) on bash platform

/PATH/hisat2-2.1.0/hisat2-build -p 8 \

Trinity.CDHITe Trinity_tran_index

/PATH/hisat2-2.1.0/hisat2 -p 8 \

-x Trinity_tran_index \

-1 data_paired.fq.gz \

-2 data_paired.fq.gz \

-S data.sam

6.Sorting the sam data

samtools(v1.10) on bash platform

/PATH/samtools view data.sam -b > data.bam

/PATH/samtools sort data.bam -o data.sorted.bam

/PATH/samtools index data.sorted.bam

1. **Annotation**

1. BUSCO analysis of the Trinity assembly

BUSCO(v.4.0.0) on bash platform

python3 /PATH/busco/src/busco/run_BUSCO.py \

-i /PATH/Trinity.CDHITe \

-o transcriptome_BUSCO \

-m transcriptome \

-l diptera_odb10 \

--config /PATH/busco/config/config.ini

2. Trinotate annotation

Annotation based on different databases on bash platform

a. pfam

/PATH/hmmscan --cpu 8 \

--domtblout TrinotatePFAM.out \

/PATH/Pfam-A.hmm \

data.transdecoder.pep > pfam.log

b. signalp(v5.0)

/PATH/signalp -format short -fasta data.transdecoder.pep

c. tmhmm(v2.0)

/PATH/tmhmm --short < data.transdecoder.pep > tmhmm.out

d. uniprot_sprot

/PATH/diamond blastx \

--db /PATH/uniprot_sprot.dmnd \

-q data \

-p 8 \

-o diamond.blastx.outfmt6

e. Drosophila coding-genes

/PATH/diamond blastx \

--db /PATH/dmel-all-translation-r6.30.dmnd \

-q data \

-p 8 \

-o fly.diamond.blastx.outfmt6

f. uniref50

/PATH/diamond blastx \

--db /PATH/uniref50.dmnd \

-q data \

-p 8 \

-o uniref50.diamond.blastx.outfmt6

g. OrthoDB.Diptera

/PATH/diamond blastx \

--db /PATH/OrthoDB.Diptera.dmnd \

-q data \

-p 8 \

-o OrthoDB.Diptera.diamond.blastx.outfmt6

h. OrthoDB.Culicidae

/PATH/diamond blastx \

--db /PATH/OrthoDB.Culicidae.dmnd \

-q data \

-p 8 \

-o OrthoDB.Culicidae.diamond.blastx.outfmt6

i. TREP database (v. 2019)

/PATH/blastn -query data \

-db /PATH/trep-db_nr_Rel-19.fasta \

-num_threads 8 \

-max_target_seqs 1 \

-perc_identity 75 \

-outfmt 6 \

-evalue 1e-3 > TE.blastn.outfmt6

1. **Differential expression analysis, principal component analysis and enrichment analysis.**

1. Estimating the abundance and building up the count matrix

Stringtie (v2.0.6) on bash platform

/PATH/stringtie-2.0.6.Linux_x86_64/stringtie \

data.sorted.bam \

-p 8 \

-G Trinity.CDHITe.transdecoder.gff3 \

-o data.assembly.gtf \

-A data.ab -B -e

2. Differential expression analysis

DESeq2 (v1.26.0) on R (v3.6.3) platform

>countData<-as.matrix(read.csv(/PATH/transcript_count_matrix.csv＂, row.names=＂transcript_id＂))

>countData<-countData[rowMeans(countData)>1,]

>condition<-factor(c(＂NB＂,＂NB＂,＂NP＂,＂NP＂))

>KIT<-factor(c("HiScanSQ","HiScanSQ","NextSeq500","HiScanSQ"))

>colData<-data.frame(row.names=colnames(countData),condition)

>all(rownames(colData) %in% colnames(countData))

>countData <- countData[, rownames(colData)]

>all(rownames(colData) == colnames(countData))

>colData$condition <- relevel(colData$condition, ＂NB＂)

>dds <- DESeqDataSetFromMatrix(countData = countData, colData = colData, design = ~ KIT+condition)

>dds <- DESeq(dds)

>normalized_counts <- as.data.frame(counts(dds, normalized=TRUE))

>contrast <- c(＂condition＂, ＂NP＂, ＂NB＂)

>res <- results(dds)

>mcols(res, use.names= TRUE)

>View(res)

3. Principal Components Analysis

stats (v3.6.2)::prcomp on R (v3.6.3) platform

>pca <- prcomp(t(normalized_counts), scale = TRUE)

4. Gene set enrichment analysis

clusterProfiler(v3.16.0)::GSEA on R (v3.6.3) platform

>GSEA(rankvalue, TERM2GENE=GO2t.CC, TERM2NAME=GO2term, pvalueCutoff = 0.05, pAdjustMethod = ＂BH＂, nPerm=10000, seed=TRUE)

5. Hypergeometric test

clusterProfiler(v3.16.0)::enricher on R (v3.6.3) platform

>enricher(gene, TERM2GENE=path2t, TERM2NAME=path2des, pvalueCutoff = 0.05, pAdjustMethod = ＂BH＂, qvalueCutoff = 0.05)

1. **Other statistical analyses**

1. F test

stats (v3.6.2)::var.test on R (v3.6.3) platform

>stat::var.test(data, alternative = "two.sided")

2. t test

stats (v3.6.2)::t.test on R (v3.6.3) platform

>t.test(data, paired=F, var.equal=F/T[depending on F test])

3. Standard error

FSA::se on R (v3.6.3) platform

>FSA::se(data)
